# MAST versus FAST for active fibrotic MASH: a meta-analysis supporting risk-stratified diagnostic pathways

**DOI:** 10.3389/fmed.2026.1779292

**Published:** 2026-04-21

**Authors:** Shengfang Liu, Qingjuan Zhang, Lijing Wang, Qin Tang, Bingtao Hu

**Affiliations:** Yibin Hospital of T.C.M., Yibin, China

**Keywords:** diagnostic accuracy, FAST, fibrosis, MASH, MAST, non-invasive diagnosis

## Abstract

**Objective:**

This meta-analysis aimed to compare the diagnostic accuracy of two new scores—FibroScan-AST (FAST) and MRI-AST (MAST)—for identifying metabolic dysfunction-associated steatohepatitis (MASH) with significant fibrosis.

**Methods:**

A systematic review and meta-analysis were conducted following PRISMA guidelines, with the protocol registered in PROSPERO. Four studies that compared FAST and MAST with liver biopsies were included. The combined sensitivity, specificity, diagnostic odds ratio (DOR), and area under the curve (AUC) were calculated using a bivariate random-effects model. Heterogeneity and threshold effects were assessed using subgroup and sensitivity analyses, respectively.

**Results:**

The FAST score showed moderate sensitivity (44.7, 95% CI: 28.3–62.4%) and quite high specificity (87.5%, 76.8–93.7%), with an AUC of 0.83 (0.75–0.89). MAST showed better sensitivity (55.9%, 41.5–69.3%) and specificity (88.1%, 85.2–90.6%), with an AUC of 0.84 (0.81–0.88). The difference in the AUC was not statistically significant (*p* = 0.964). Compared to FAST, MAST showed a relatively higher positive likelihood ratio (4.71 vs. 3.54) and DOR (9.42 vs. 5.68), although these differences were not statistically significant. FAST showed significant threshold variability (*p* = 0.005), whereas MAST results were more consistent.

**Conclusion:**

Although MAST and FAST differ in sensitivity and stability, their overall diagnostic accuracy does not differ statistically. The core limitation of this study is that only four studies were included, which restricts the reliability of the pooled estimates. Therefore, the findings should be interpreted with caution.

**Systematic review registration:**

https://www.crd.york.ac.uk/PROSPERO/view/CRD420251109029.

## Introduction

Metabolic dysfunction-associated steatotic liver disease (MASLD) is currently the leading cause of chronic liver disease worldwide. MASLD progresses from simple hepatic steatosis to active fibrotic metabolic dysfunction-associated steatohepatitis (MASH), which is defined as an NAFLD Activity Score (NAS) ≥ 4 and fibrosis stage ≥ F2. This progression significantly increases the risk of cirrhosis and hepatocellular carcinoma (1–3). Although liver biopsy remains the diagnostic gold standard, it is invasive, expensive, and can yield discordant results due to sampling variability; therefore, it is not widely used. Although non-invasive alternatives (such as serum biomarkers and imaging-based scores) have improved, the diagnostic accuracy, robustness, and clinical utility of these tools remain subjects of debate (4, 5). A major challenge is balancing sensitivity and specificity to reliably identify high-risk patients while avoiding unnecessary biopsies. Globally, MASLD affects approximately 25–30% of the general population (equivalent to more than 1 billion people) (6), of whom 20% will progress to MASH. However, the rate of liver biopsy utilization remains below 5%, highlighting the urgent need for reliable non-invasive diagnostic approaches in MASLD management (7–17).

Recent improvements in elastography and biomarker-based scoring systems, such as the FibroScan-AST (FAST) and MRI-AST (MAST) scores, have introduced new ways to identify MASH (18–20). The FAST score integrates three key parameters, liver stiffness measurement (LSM), controlled attenuation parameter (CAP), and serum aspartate aminotransferase (AST) levels, which are specifically designed to detect MASH, defined as NAS ≥ 4 and fibrosis ≥ F2 (21). FAST scoring is easy to perform in clinical practice but may show significant differences depending on the thresholds used. Furthermore, elastography-based methods (including FibroScan for FAST score) have inter-observer variation and are affected by body mass index (BMI); higher BMI is associated with increased abdominal fat thickness, which may compromise the accuracy of LSM and CAP measurements. Conversely, the MAST score combines MRI-proton density fat fraction (MRI-PDFF), magnetic resonance elastography (MRE) stiffness values, and AST levels (22). Although originally developed to achieve higher diagnostic accuracy in the referred population, this score requires dedicated MRI equipment, is more costly, and has limited accessibility. There is a significant cost difference between the two technologies: FibroScan is relatively low-cost and portable, whereas the MRI equipment required by MAST is expensive and less accessible, especially in resource-scarce environments.

Although each score has potential applications, most previous studies have evaluated only one score separately, resulting in limited direct comparative evidence. Furthermore, differences in study populations (such as geographic area and BMI distribution) and inconsistent thresholds across studies hinder clinical transformation. Therefore, a rigorous evaluation of the diagnostic performance of the FAST and MAST scores is essential to clarify their respective roles in patient stratification (19, 20, 23, 24). Importantly, no prior meta-analysis has directly examined FAST and MAST scores for diagnosing active fibrotic MASH, nor has it addressed the confusing effects of population and methodological differences. To fill this gap, we conducted a systematic review and meta-analysis with goals to (1) measure and compare the accuracy of both scores in diagnosis, (2) identify sources of heterogeneity, such as geographic region or BMI, and (3) preliminarily explore the performance differences between the two scoring systems across various clinical scenarios (such as different BMI categories and regions), and to provide hypotheses for future larger-scale studies.

## Methods

### Review protocol registration

This review protocol was registered in the PROSPERO database (International Prospective Register of Systematic Reviews; registration number: CRD420251109029).

### Search strategy and selection criteria

We conducted a systematic literature search in PubMed/MEDLINE, Web of Science Core Collection, Embase, Scopus, and the Cochrane Library up to May 30, 2025, using a dual-strategy method that incorporated controlled vocabularies (MeSH and Emtree terms) with free-text keywords (for example, ‘non-alcoholic steatohepatitis,’ ‘MASH,’ ‘MASH fibrosis,’ ‘NASH,’ ‘NASH fibrosis,’ ‘fibrosis,’ ‘FAST score,’ ‘MAST score,’ and ‘non-invasive diagnosis’). Two independent researchers (S. L. and L. W.) performed a two-step screening process: an initial title and abstract review, followed by a full-text assessment of potentially eligible studies. Discrepancies were resolved through consensus arbitration by a senior author (B. H.), ensuring strict adherence to the predefined inclusion criteria during study selection.

To be included in this meta-analysis, studies had to meet the following five set criteria: (1) they must enroll adult patients (≥18 years old) with biopsy-proven MASLD; (2) the study should focus on active fibrotic MASH, which is defined according to the histological criteria widely used in the FLINT trial and subsequent MASH clinical studies, namely NAFLD activity score (NAS) ≥ 4 and fibrosis stage ≥ F2 (25). (3) There must be a direct comparison of the FAST score (which includes serum AST, FibroScan liver stiffness measurement, and controlled attenuation parameter) and the MAST score (which combines magnetic resonance elastography with serum AST) in the same group of patients, specifically examining high-specificity cutoffs used in clinical practice to confirm fibrosis rather than ruling it out; (4) full reporting of diagnostic performance metrics for both scoring systems in detecting fibrosis; and (5) use of liver biopsy histopathology as the gold standard, with cutoff choices based on a review of the literature and current clinical guidelines to ensure that the method is consistent and relevant to real-world use.

Studies were excluded from the meta-analysis based on the following criteria: (1) non-original research papers, such as case reports, review articles, editorials, and conference abstracts (unless they provided complete data); (2) absence of direct comparison data for both FAST and MAST scores; (3) inclusion of patient groups with other liver diseases (such as viral hepatitis, autoimmune hepatitis, or alcoholic liver disease) or children under 18 years of age; and (4) use of inadequate reference standards. This systematic review and meta-analysis were conducted in accordance with the PRISMA guidelines^1^ to ensure a clear, reproducible methodology.

### Data extraction and quality assessment

Two researchers (S. L. and L. W.) extracted data from all studies, and the following information was collected: study authors, year of publication, study design, patient characteristics (e.g., age, sex, BMI), cutoff values for the FAST and MAST scores, and their diagnostic performance, including sensitivity, specificity, AUROC, and 95% confidence intervals. The quality of the included studies was assessed using the QUADAS-2 tool by two independent reviewers (S. L. and L. W.) who were blinded to the study details, and they evaluated potential biases in patient selection, blinding of the index tests and reference standards, and patient flow. Any disagreements between the reviewers were resolved through discussions with a senior researcher (B. H.) to ensure consistency in the quality assessment.

### Data synthesis and analysis

We conducted a bivariate meta-analysis to evaluate and compare the diagnostic performance of the FAST and MAST scores in identifying active fibrotic MASH. MASH was defined as an NAS score ≥ 4 and fibrosis stage F2 or higher. We examined the sensitivity, specificity, and AUROC. One modification was made after the protocol registration. Specifically, the planned individual studies would be the unit of analysis. However, the study by Imajo et al. (24) contained data from three distinct cohorts. To maintain data independence and assess the performance of each cohort individually, we treated these groups as separate units in the final meta-analysis. Therefore, six datasets were included.

### Statistical analysis

To evaluate potential threshold effects and the impact of small sample sizes, we planned the following analytical approaches: (1) assessment of threshold effects using Spearman’s correlation between logit-transformed sensitivity (Se) and specificity (Sp), and (2) evaluation of small-study effects through leave-one-out sensitivity analysis (26, 27). Our primary analysis employed a bivariate random-effects model to pool the diagnostic accuracy of the FAST and MAST scores for detecting active fibrotic MASH (defined as NAS ≥ 4 plus fibrosis stage≥F2) with hierarchical summary ROC curve modeling (26, 27). To ensure appropriate model fit, a continuity correction was applied (adding 0.5 to all true positives, false positives, false negatives, and true negatives) before calculating diagnostic parameters such as transformed Se, Sp, and diagnostic odds ratios (DORs) (26, 27).

We examined the threshold effects using Spearman’s rank correlation and linear regression of logit(Se) against logit(1-Sp). The Metandi method uses maximum likelihood estimation for the bivariate model, generating pooled Se and Sp estimates with 95% confidence intervals, curve parameters, and area under the curve (AUC) values, while also accounting for the Se-Sp covariance structure. Between-study heterogeneity was measured using Cochran’s Q statistic (*p* < 0.05 indicating significant heterogeneity). For studies demonstrating significant heterogeneity, subgroup analyses were performed according to the study design (prospective cross-sectional or retrospective cohort), geographic region (Asian or Western populations), and adiposity status (BMI ≤ 28 or >28 kg/m^2^).

Owing to the limited number of studies included, this study did not formally evaluate publication bias, and all subgroup analyses were predefined as exploratory analyses (26, 27). All statistical analyses were performed using STATA 17.0 with specialized meta-analysis packages, including Metandi, Metan, and Midas. Sensitivity analysis was conducted by sequentially excluding each study to assess the robustness of our primary results across different models (26, 27).

## Results

### Study inclusion

**Figure 1:**
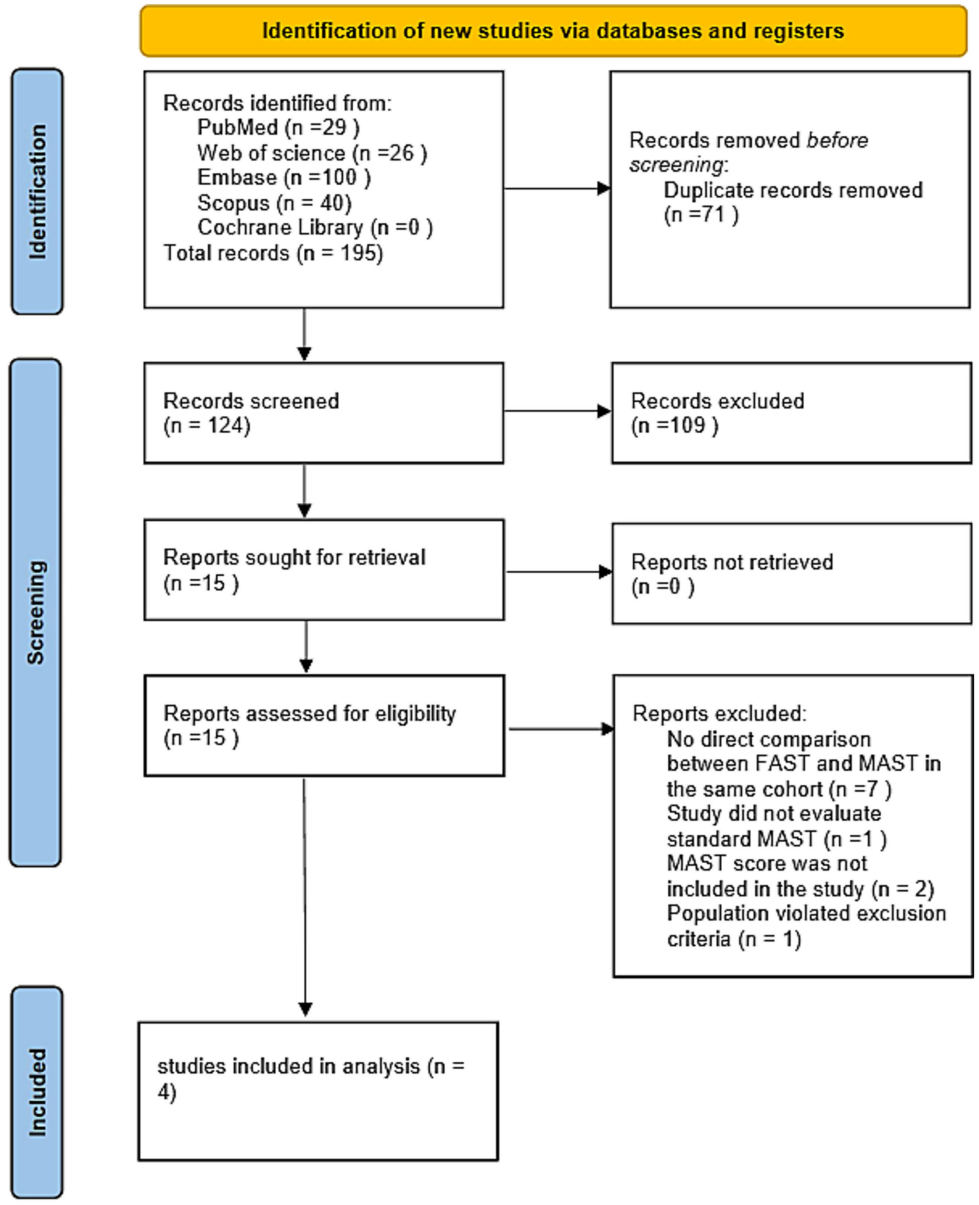
Literature screening flowchart. Process of identifying and screening study records through various databases, including deduplication, exclusion, and the final number of studies included in the analysis.

The process by which we identified the studies for the meta-analysis is shown in Figure 1. Our search identified 195 potential records, from which we removed 71 duplicates, leaving 124 for initial screening. After reviewing titles and abstracts, we retrieved 15 full-text articles for a full check. Then we applied our inclusion criteria, and nine papers were excluded due to methodological issues (details in Figure 1). In the end, four studies were included in the quantitative synthesis. The whole process of selecting studies, with why we left some out at each step, is shown in the PRISMA flow chart (Figure 1), which clearly shows the progression from finding studies first to putting them in our final meta-analysis.

### Study characteristics

Our meta-analysis used data from four observational studies. It is important to note that Imajo et al. (24) reported data from three distinct cohorts; each cohort had its own population sources, independent diagnostic cutoffs, and individual contingency tables. To ensure statistical independence and preserve the diagnostic performance characteristics of each cohort, we treated them as separate units for analysis. Therefore, the final analysis had six datasets (three cohorts from Kento et al., plus one cohort from each of the other three studies). This method helped to show the variability at the population level in the literature and strengthened the analysis. The four included studies used the same histological definition of active fibrotic MASH (NAS ≥ 4 and fibrosis stage ≥F2) to ensure consistency of the target disease definition across cohorts. A summary of the histological definitions used in each study is provided in Supplementary Table S1.

The FAST diagnostic thresholds were set at 0.65, 0.47, 0.67, and 0.67, whereas the MAST score thresholds ranged from 0.159 to 0.26. Although FAST thresholds varied considerably (0.47–0.67), with the lowest value (0.47) approaching the previously established exclusion criterion (0.35), we retained all thresholds to avoid selection bias and to maintain enough statistical power, given the limited sample sizes. This approach was subsequently validated through comprehensive threshold-effect and sensitivity analyses (see Results), ensuring the use of all available data while maintaining methodological rigor. Furthermore, the choice to maintain all thresholds was further supported by their clinical relevance and alignment with established diagnostic confirmation goals in MASH evaluation.

Table 1 shows the basic characteristics of the included studies. Three studies included participants from Asia (China, Korea, and Japan), one study was conducted in the United States, and one additional group in Imajo et al. (24) was from the United States. Three studies used cross-sectional designs, but one used a case-cohort approach. In the Kento 2023b group, because some vibration-controlled transient elastography (VCTE) or controlled attenuation parameter (CAP) data were incomplete, FAST scores were available for 122 out of 169 patients, and the basic factors (e.g., sex, BMI, age) were not described separately. Conversely, magnetic resonance elastography-based MAST scores were present for all participants. In total, 1,047 biopsy-confirmed MASLD patients were examined, and fibrotic MASH (NAS ≥ 4 plus fibrosis stage ≥F2) was observed in 11.5 to 50.8% of the different groups. These cohorts exhibited typical metabolic profiles: average age 35–61.8 years, mostly men (13.6–59.8%), and BMI 27.7–33.3 kg/m^2^.

**Table 1:** Main characteristics of the included studies.

Author	ID	Country	Study Type	Gold Standard	Scoring Tool	Threshold (Cut-off)	Total Subjects, n	Fibrotic MASH, n (%)	Sex, Men n (%)	Age	BMI
Qi (2024) (20)	1	China	Cross-sectional	Liver biopsy	FAST	0.65	108	28 (25.9%)	60 (55.6%)	38 ± 12.6	28.7 ± 4.1
					MAST	0.159					
Choi (2024) (23)	2	Korea	Cross-sectional	Liver biopsy	FAST	0.47	116	16 (13.8%)	38 (32.8%)	35 ± 12	33.3 ± 6.5
					MAST	0.26					
Noureddin (2022) (19)	3	USA	Case-cohort	Liver biopsy	FAST	0.67	244	28 (11.5%)	146 (59.8%)	55.7 ± 6.3	33.1 ± 5.1
					MAST	0.242					
Imajo (2023) (24)	4	Japan	Cross-sectional	Liver biopsy	FAST	0.67	176	78 (44.3%)	102 (58%)	60.9 ± 13.0	28.4 ± 4.59
					MAST	0.242					
Imajo (2023) (24)	5	Japan	Cross-sectional	Liver biopsy	FAST	0.67	122	62 (50.8%)	–	–	–
					MAST	0.242	169	73 (43.2%)	23 (13.6%)	61.8 ± 15.5	27.7 ± 4.86
Imajo (2023) (24)	6	USA	Cross-sectional	Liver biopsy	FAST	0.67	234	46 (19.7%)	103 (44%)	52.4 ± 13.1	31.7 ± 4.48
					MAST	0.242					

FAST: FibroScan-AST (Combined score of liver stiffness measurement by FibroScan and AST level for identifying fibrotic MASH). MAST: MRI-AST (Combined score of MRI-proton density fat fraction and AST level for identifying fibrotic MASH). MASH: Metabolic dysfunction-associated steatohepatitis. BMI: Body mass index (kg/m^2^).

### Study quality assessment

**Figure 2:**
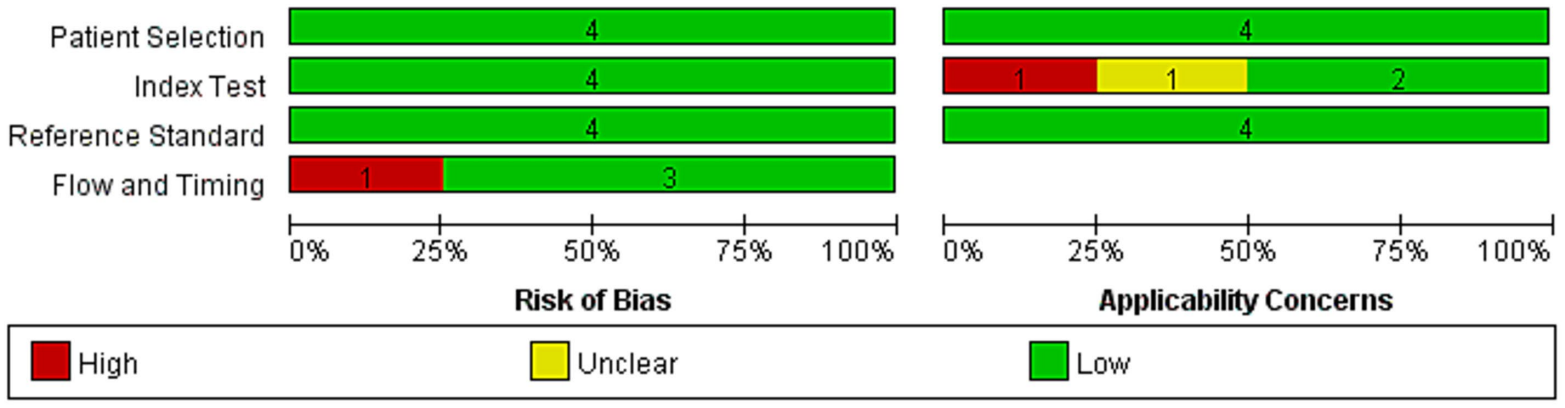
Summary of risk of bias assessment. Risk of bias and applicability concerns of included studies assessed using the QUADAS-2 tool.

We assessed the methodological quality of the four studies (19, 20, 23, 24) with the QUADAS-2 tool. As shown in Figure 2, the bias risk varied across domains. Most domains in the studies were rated as low-risk, but some were rated as unclear. For patient selection, all studies exhibited a risk of bias: two studies (50%) were at high risk because they did not adequately describe how patients were enrolled. The other two (50%) were at unclear risk because the reports were incomplete. For the index test, three studies (75%) demonstrated low risk because they used standardized FAST and MAST assessments, and one study (25%) had unclear risk because the methodological details were inadequate. All four studies (100%) achieved a low risk in the reference standard domain because liver biopsies were evaluated without knowledge of other test results. Regarding flow and timing, three studies (75%) were at low risk because they clearly specified the interval between the biopsy and the other test, whereas one study (25%) had unclear risk because the information was insufficient. Overall, the reference standard domain was the most robust, but patient selection was the main area that required improvement. One study (25%) was of high quality; however, three studies (75%) had methodological limitations. These results indicate that future studies are needed to enhance clarity in patient selection procedures and the timing of assessments to improve reliability.

### Meta-analysis of FAST score diagnostic performance

**Figure 3:**
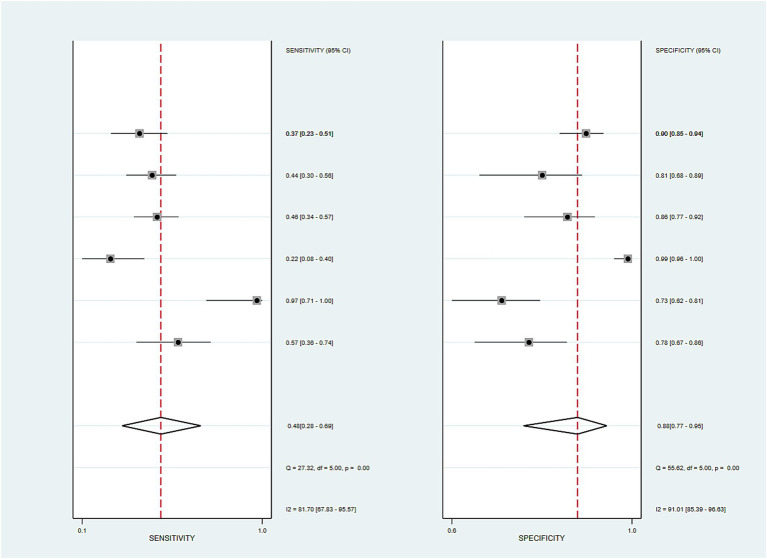
Forest plot of sensitivity and specificity for the FAST score. This figure presents the sensitivity and specificity of the FAST score with 95% confidence intervals for each included study. Pooled estimates were derived using a random-effects model. Heterogeneity tests revealed a *Q* statistic of 27.32 (*p* < 0.01) with an I^2^ of 81.70% for sensitivity, and a *Q* statistic of 55.62 (*p* < 0.01) with an I^2^ of 91.01% for specificity, indicating substantial heterogeneity among studies.

As shown in the forest plot (Figure 3), significant heterogeneity was observed in the diagnostic performance of the FAST score for fibrotic MASH. Sensitivity demonstrated marked between-study variation (*I*^2^ = 81.7%, *p* < 0.001), ranging from 0.22 (95% CI: 0.08–0.4) to 0.97 (95% CI: 0.71–1.00). Extreme heterogeneity (*I*^2^ > 80%) compromised the reliability of the pooled sensitivity estimates, suggesting that diagnostic sensitivity may be influenced by population characteristics and cutoff selection. Specificity analysis similarly showed high heterogeneity (*I*^2^ = 91.01%, *p* < 0.001), though values remained generally elevated (0.73–0.99), with 4/6 studies exceeding 0.8 and Study 4 reaching 0.99 (95% CI: 0.96–1.00). The pooled specificity (0.88) supports its potential as a confirmatory tool; however, considerable between-study differences necessitate individualized clinical interpretation. This extreme heterogeneity (I^2^ > 80%) indicates that the diagnostic performance of the FAST score varies substantially across studies. The combined sensitivity (44.7%) and specificity (87.5%) we calculated were only summary estimates and could not represent stable performance across all clinical scenarios.

**Figure 4:**
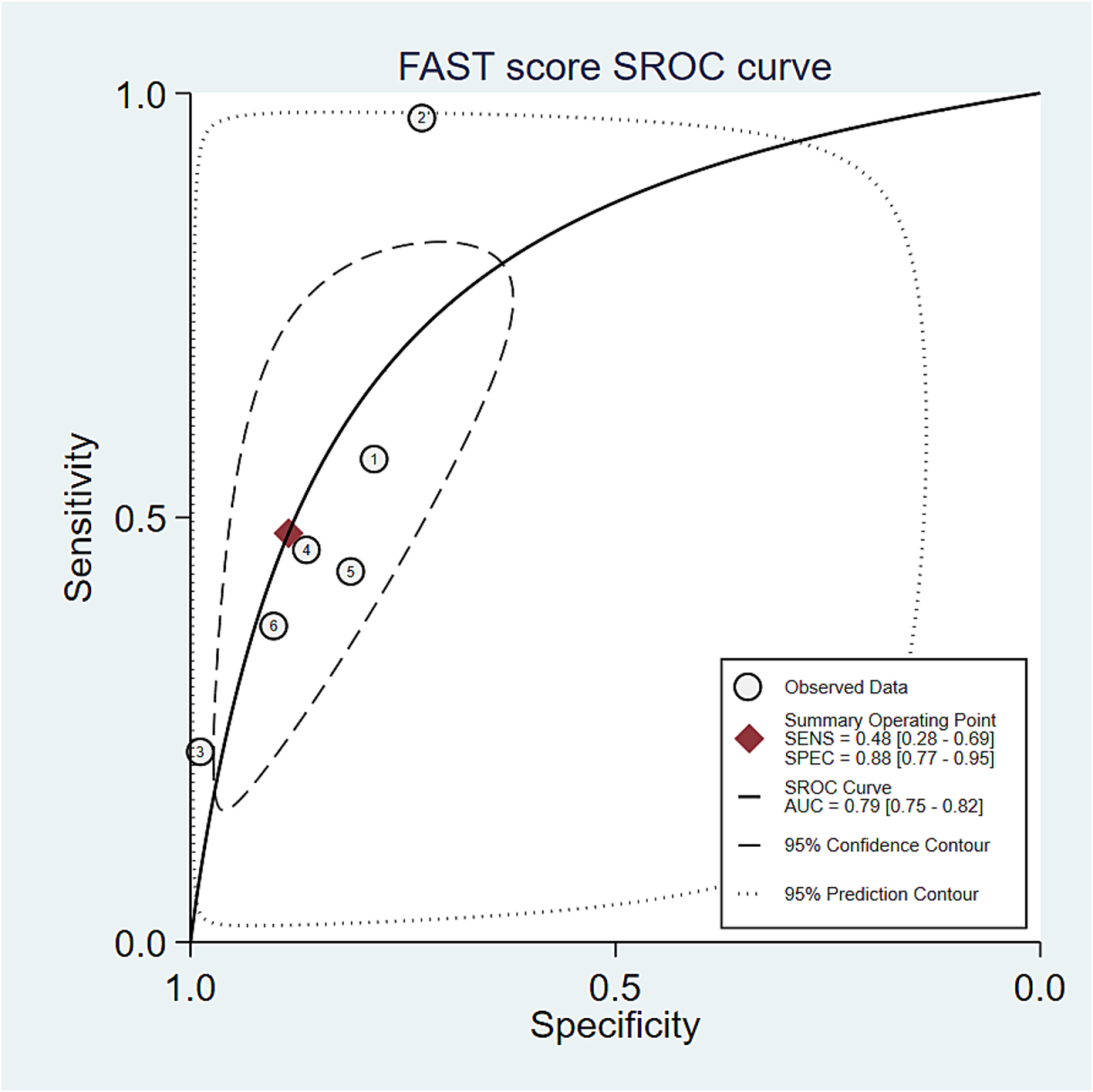
Summary receiver operating characteristic (SROC) curve for FAST score. Legend: SROC curve showing pooled sensitivity and specificity. AUC = 0.79 [0.75–0.82]. Shaded areas represent the 95% confidence and prediction intervals.

Bivariate mixed-effects modeling confirmed moderate diagnostic accuracy for active fibrotic MASH. The combined numbers were: sensitivity of 0.447 (95% CI: 0.283–0.624), specificity of 0.875 (95% CI: 0.768–0.937), and DOR of 5.682 (95% CI: 3.622–5.913). The positive likelihood ratio (LR+) was 3.537 (95% CI: 2.392–5.380), which means it provides some useful diagnostic information for positive findings, but the negative likelihood ratio (LR-) was 0.631 (95% CI: 0.481–0.813), which indicates limited ability to exclude the disease. The AUC of the model was 0.827 (95% CI: 0.782–0.872). The summary receiver operating characteristic (SROC) curve analysis (Figure 4) yielded a combined sensitivity of 0.48 (95% CI: 0.28–0.69), specificity of 0.88 (95% CI: 0.77–0.95), and AUC of 0.79 (95% CI: 0.75–0.82). These results support the overall usefulness for diagnosis.

Clinically, the FAST score showed a moderate positive likelihood ratio (LR + = 3.537) and specificity. However, the negative predictive value (NPV) is highly dependent on disease prevalence and cannot be reliably estimated in a meta-analysis; therefore, this study reported the negative likelihood ratio (LR − = 0.631) and not a fixed combined negative predictive value. Furthermore, the sensitivity of the FAST score was poor (0.447), and there was significant heterogeneity. Caution is needed when interpreting the results in the “gray zone” near the diagnostic threshold (26). The overall accuracy of this score was medium (AUC = 0.79), suggesting that the FAST score can be used in combination with other noninvasive examinations to obtain more reliable and accurate diagnostic results.

### Meta-analysis of MAST score diagnostic performance

**Figure 5:**
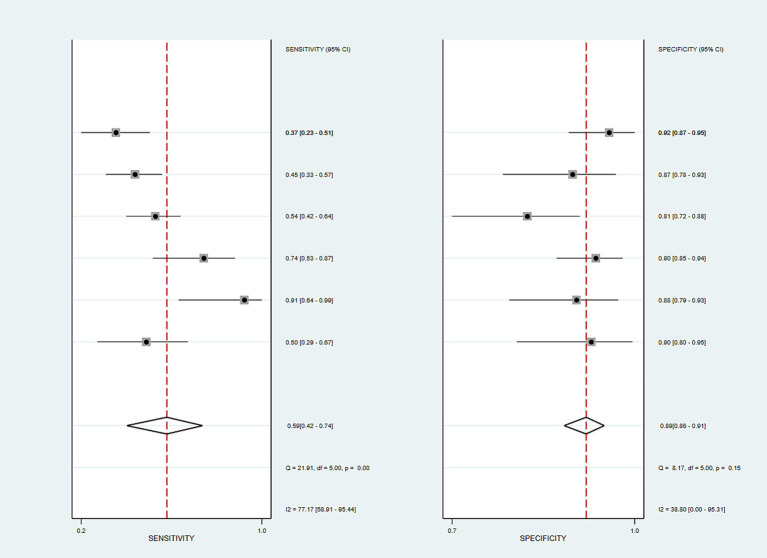
Forest plot of sensitivity and specificity for the MAST score. The figure shows the estimated sensitivity and specificity of the MAST score for assessing diagnostic accuracy, along with the 95% confidence interval in each study. The random-effects model was used to calculate the pooled estimate, with a pooled sensitivity of 0.59 (95% confidence interval: 0.42–0.74) and a pooled specificity of 0.89 (95% confidence interval: 0.86–0.91). Significant heterogeneity was observed in the pooled sensitivity analysis (*Q* = 21.91, *p* < 0.01; *I*^2^ = 77.17%) and moderate heterogeneity in the pooled specificity analysis (*Q* = 8.17, *p* = 0.15; *I*^2^ = 38.80%).

Forest plot analysis showed that the MAST score had moderate sensitivity (pooled sensitivity: 0.59, 95% CI: 0.42–0.74) and high specificity (pooled specificity: 0.89, 95% CI: 0.86–0.91) for diagnosing fibrotic MASH. The heterogeneity of sensitivity was relatively high (*I*^2^ = 77.17%, *p* < 0.001; Figure 5), indicating inconsistent sensitivity across results. However, heterogeneity in specificity was low (*I*^2^ = 38.8%, *p* = 0.15; Figure 5), indicating that the index was more stable and reliable in excluding non-patients. Although the specificity of the MAST is relatively stable, the high heterogeneity in sensitivity indicates that the test’s ability to correctly identify patients varies significantly in different cohorts, which also limits our confidence in the combined estimate.

**Figure 6:**
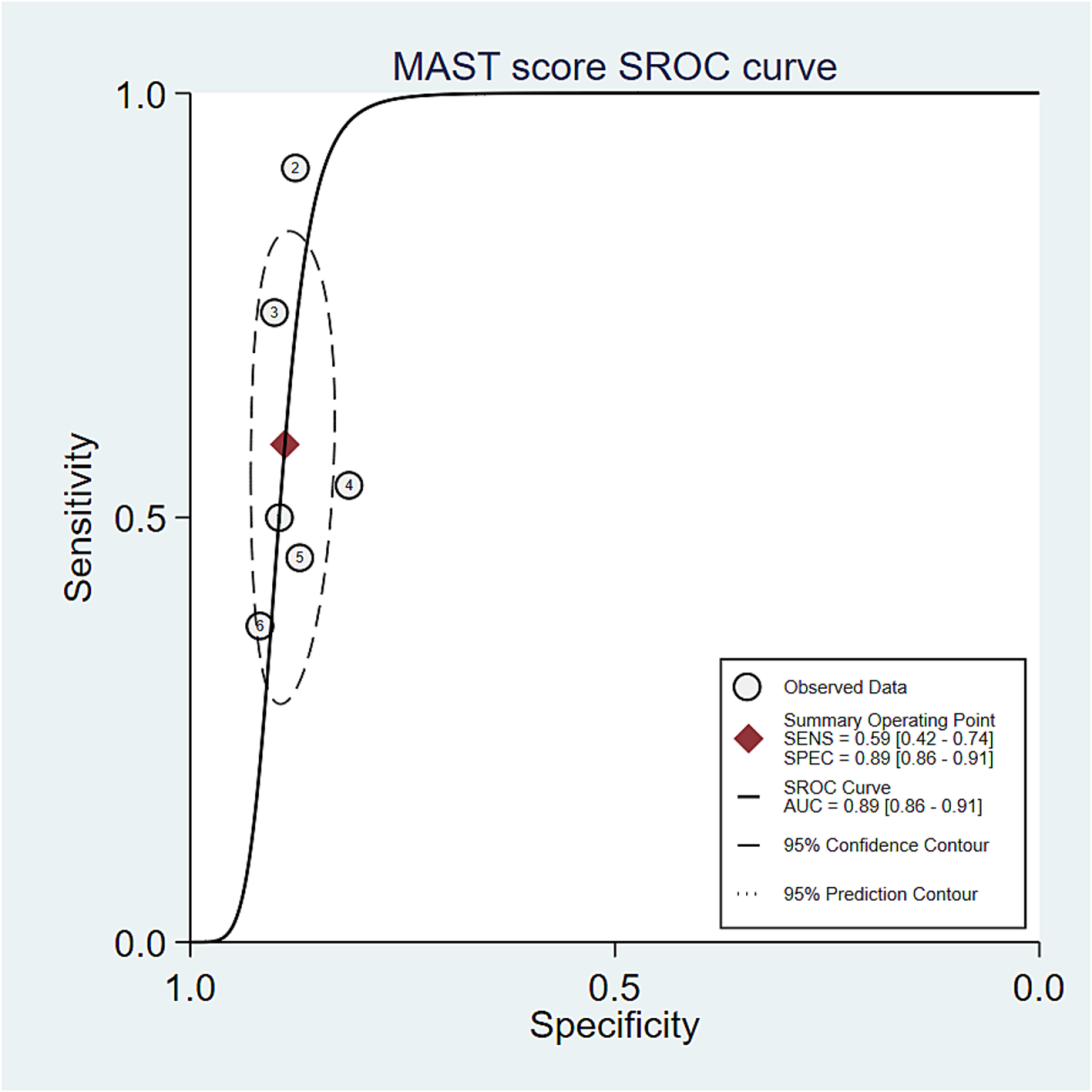
Summary receiver operating characteristic (SROC) curve for MAST score. Legend: SROC curve showing pooled sensitivity and specificity. AUC = 0.89 [0.86–0.91]. Shaded areas represent the 95% confidence and prediction intervals.

The bivariate model showed that the MAST score was useful for identifying fibrotic MASH. Its pooled sensitivity (0.559; 95% CI: 0.415–0.693) was better than that of the FAST score (0.447), and it had similar specificity (0.881 vs. 0.875). The MAST score had a higher DOR (DOR = 9.419; 95% CI: 5.151–17.224) and positive likelihood ratio (LR + =4.715; 95% CI: 3.422–6.497) compared to the FAST score (DOR = 5.682; LR + =3.537), which means it can tell the difference better and has a better positive predictive value. Moreover, its lower negative likelihood ratio (LR − =0.509; 95% CI: 0.363–0.690) vs. FAST (0.631; 95% CI: 0.490–0.812) indicates that it is better at ruling out the disease. The AUC from the bivariate model (AUC = 0.843; 95% CI: 0.801–0.885) and the SROC curve analysis (AUC = 0.89; 95% CI: 0.86–0.91: Figure 6) collectively demonstrated that it had high overall diagnostic accuracy.

We examined diagnostic performance using three methods: bivariate mixed-effects modeling, summary receiver operating characteristic analysis, and traditional univariate meta-analysis. All methods yielded generally consistent results, but some small differences in the numbers were observed. This is probably because of how they handle differences between studies; bivariate models assess sensitivity and specificity together, whereas univariate approaches analyze them separately. Forest plots showed small differences between what each study found and what the models said, highlighting the impact of analytical methods on pooled estimates. Given that bivariate models are methodologically preferred for diagnostic meta-analyses, we believe that their results should be used first.

### Comparison of diagnostic performance between the MAST score and the FAST score

Table 2 shows that the comparative analysis identified differences in diagnostic performance features between MAST and FAST scores for finding fibrotic MASH. MAST had a slightly higher sensitivity (55.9% vs. 44.7%; difference: 0.079, *p* = 0.392) and a higher DOR (DOR: 9.419 vs. 5.682; difference: 3.74, *p* = 0.269), but these differences were not statistically significant. Both scores had similar specificities (88.1% vs. 87.5%, *p* = 0.779) and areas under the curve (AUC: 0.84 vs. 0.83, *p* = 0.964) (28). MAST showed a higher positive likelihood ratio (4.71 vs. 3.54) and a lower negative likelihood ratio (0.509 vs. 0.631), but these differences were not statistically significant. To further verify the difference in diagnostic effectiveness between the two scoring systems, we conducted an interaction test based on a meta-regression. The results showed no statistically significant interaction between FAST and MAST scores for sensitivity (*P* for interaction = 0.393) or specificity (*P* for interaction = 0.725).

**Table 2:** Comparison of diagnostic accuracy between FAST and MAST scores using a bivariate model.

Metric	FAST (Adjusted)	MAST (Adjusted)	Difference (FAST - MAST)	Statistical Test (Z-value)	*p*-value
Sensitivity	0.447 (0.283–0.624)	0.559 (0.415–0.693)	−0.078	−0.856	0.392
Specificity	0.875 (0.768–0.937)	0.881 (0.852–0.906)	−0.013	−0.281	0.779
DOR	5.682 (3.622–8.913)	9.419 (5.151–17.224)	−3.737	–	0.433
LR+	3.537 (2.392–5.380)	4.715 (3.422–6.497)	−1.178	–	–
LR-	0.631 (0.481–0.812)	0.509 (0.363–0.690)	0.122	–	–
AUC	0.827	0.843	−0.016	−0.045	0.964

FAST: FibroScan-AST (Combined score of liver stiffness measurement by FibroScan and AST level for identifying fibrotic MASH). MAST: MRI-AST (Combined score of MRI-proton density fat fraction and AST level for identifying fibrotic MASH). AUC: Area Under the ROC Curve. DOR: Diagnostic Odds Ratio. LR+: Positive Likelihood Ratio. LR–: Negative Likelihood Ratio. Values in parentheses represent 95% confidence intervals. *Z*-values were used to test the significance of differences between the FAST score and MAST score groups. *p*-value <0.05 indicates statistical significance.

### Heterogeneity analysis

Heterogeneity analysis identified a significant threshold effect in the FAST group (Spearman’s *ρ* = 0.943, *p* = 0.005; Beta = 0.862, Lambda = 13.536), driven primarily by differences in cutoff value selection across studies; the MAST group showed no significant threshold effect (Spearman ρ = 0.257, *p* = 0.623) and better inter-study consistency. The two groups presented significant differences in the HSROC model parameters. The FAST group had a Lambda of 1.593 (95% CI: 0.641–2.545) and a Theta of −1.023 (95% CI: −1.752 to −0.293), whereas the MAST group had a Lambda of 4.024 (95% CI: −0.575–8.624) and a Theta of −1.891 (95% CI: −4.312–0.530). These differences were also reflected in the SROC curves, which showed a higher dispersion of data points for the FAST group and a more concentrated distribution for the MAST group. Bivariate model results demonstrated significantly higher inter-study variances for logit-transformed sensitivity (logitSe: 0.594, 95% CI: 0.103–3.441) and logit-transformed specificity (logitSp: 0.782, 95% CI: 0.198–3.092) in the FAST group, which showed a correlation coefficient of −1 and a covariance of −0.114. This resulted in more pronounced heterogeneity-related biases in diagnostic performance, thereby confirming extremely high heterogeneity in both sensitivity and specificity for the FAST group, with significantly lower heterogeneity observed for the MAST group. Meta-regression analysis showed no significant group effect on logDOR, with pooled DOR values of 5.682 for the FAST group and 9.419 for the MAST group, respectively; the intergroup heterogeneity was Cochran’s Q = 1.22, df = 1, *p* = 0.269. No independent variables showed significant effects in the regression models, indicating that the included variables did not adequately explain the observed heterogeneity.

### Subgroup analysis results

We conducted an exploratory subgroup analysis to generate hypotheses for future research; however, due to the limited number of included studies, these findings are limited. Subgroup analyses (Table 3) showed small performance differences between FAST and MAST scores of the different groups. Among Asian groups, the AUC difference was negligible (ΔAUC = − 0.017, *p* = 0.961). In moderate/low-quality studies (QUADAS-2 score ≤ 2), the FAST score had slightly higher diagnostic accuracy (AUC: 0.85 vs. 0.84, *p* = 0.999). Similarly, in an analysis restricted to studies with a mean BMI > 28 kg/m^2^, the MAST score showed a numerically higher AUC, but the difference was not statistically significant (ΔAUC = −0.020, *p* = 0.962) (29).

**Table 3:** Exploratory Subgroup Analysis: FAST vs. MAST Group Comparisons.

Subgroup Variable	Group	AUC (Adjusted)	AUC Difference (FAST - MAST)	*Z*-value	*p*-value
Region (Asia)	FAST	0.8238	−0.0169	−0.0493	0.9607
	MAST	0.8407			
Quality (Low/Moderate)	FAST	0.8479	0.0118	0.0016	0.9987
	MAST	0.8361			
BMI (>28)	FAST	0.8292	−0.0195	−0.0477	0.9620
	MAST	0.8488			

FAST: FibroScan-AST (Combined score of liver stiffness measurement by FibroScan and AST level for identifying fibrotic MASH). MAST: MRI-AST (Combined score of MRI-proton density fat fraction and AST level for identifying fibrotic MASH). AUC: Area Under the ROC Curve, indicating diagnostic accuracy. BMI: Body Mass Index, >28 is defined as obesity. Region: Subgroup analysis by region, Asia refers to the Asian population. Quality: Subgroup analysis by study quality, Low/Moderate refers to low to moderate-quality studies. AUC values are adjusted estimates, with 95% confidence intervals in parentheses. *Z*-values test the significance of AUC differences between FAST and MAST scores across subgroups. *p*-value <0.05 indicates statistical significance. These subgroup analyses are based on a small number of studies and data sets. The results should be considered exploratory and hypothesized, because the analysis is statistically underpowered and the results may be unstable. They need to be verified in larger, future cohorts.

### Sensitivity analysis and assessment

The FAST group showed sensitivity from 0.47 to 0.54, specificity from 0.86 to 0.90, and AUC from 0.83 to 0.84. When the study with a cutoff of 0.47 was excluded, sensitivity improved from 44.7 to 48.1%, but specificity decreased from 87.5 to 85.3%, and the AUC change was minimal (less than 0.01). With only four included studies, leave-one-out sensitivity analyses revealed no dominant effect of any single study (AUC fluctuation < 0.02). The MAST group showed sensitivity between 0.45 and 0.58, specificity between 0.85 and 0.92, and AUC between 0.83 and 0.85. Following the removal of any single study, the change in AUC remained < 0.02. Sensitivity analysis indicated robust results for both groups (AUC fluctuation < 0.02), and the primary findings were consistent across subgroups, including Asian populations or different BMI levels. After removal of high-quality studies, FAST specificity dropped to 85.5% (95% CI: 81.3–88.9%), whereas sensitivity increased to 53.7%. The pooled MAST AUC was 0.836, with a slight 0.007 difference from the overall AUC of 0.843, indicating no notable changes. The MAST score maintained consistent diagnostic performance across single-study removals and study-quality stratifications, confirming dependable results.

## Discussion

This meta-analysis presents a systematic review comparing the diagnostic performance of the FAST and MAST scores in identifying active fibrotic MASH with NAS ≥ 4 and fibrosis stage ≥ F2 (25), with no prior direct comparative meta-analysis available (30–32, 36). Using a bivariate model analysis of six independent cohorts, we demonstrated that the MAST score exhibits slightly better overall diagnostic accuracy than the FAST score (18–20, 24, 33).

The MAST score demonstrated better sensitivity and overall diagnostic accuracy for fibrotic MASH than the FAST score, while maintaining similar specificity. Importantly, its lower heterogeneity, especially in specificity, indicated greater stability, which supports its potential application in clinical settings (18–20, 33). However, the direct comparison between the MAST and FAST scores was not statistically significant; therefore, further validation in larger, independent cohorts is needed to confirm these results. In particular, the heterogeneity in sensitivity and specificity for the FAST score was over 80%. This extreme heterogeneity suggested that pooled estimates may not reflect their true performance across all populations.

Heterogeneity analysis confirmed that a significant threshold effect in the FAST group was the main source of within-group heterogeneity, whereas the MAST group presented no significant threshold effect and better inter-study consistency. The FAST group had significantly higher logitSe and logitSp inter-study variances and DL method-based heterogeneity indicators than the MAST group, and the SROC curve visually reflected the higher dispersion of the research points in the FAST group. Meta-regression analysis showed that neither group allocation nor independent variables such as demographic and research characteristics could adequately explain inter-study heterogeneity, suggesting that the heterogeneity may stem from unmeasured or unincluded potential factors, and only study quality was a potential influencing factor for sensitivity differences. Subgroup analysis revealed no statistically significant between-group differences in diagnostic performance in the Asian population or in subgroups stratified by BMI and study quality, whereas the generalizability of the findings was limited because most studies were from Asia. Sensitivity analysis confirmed the robustness of the study results, with AUC fluctuations for both scores being below 0.02 after excluding any single study, and the MAST group showed better overall stability in diagnostic efficacy.

Spectrum bias may have influenced the present findings. Most enrolled patients were from tertiary referral centers with high rates of advanced fibrosis, and application to primary care populations with lower disease prevalence may lead to overestimation of diagnostic accuracy and restrict generalizability. The diagnostic performance of FAST and MAST scores reported in this article is derived from the original research, which used high-specificity thresholds of FAST ≥0.65–0.67 and MAST ≥0.159–0.26. These high-specificity thresholds were designed to diagnose active fibrotic MASH in high-risk populations. This approach aligns with the need for precise identification of patients requiring targeted therapeutic intervention and specialized management; therefore, the results of this study were most suitable for confirmatory diagnostic clinical settings. The included studies did not provide complete low-threshold diagnostic data, and there was no uniform standard for low-threshold selection across studies. Given the substantial heterogeneity, forcibly pooling low-threshold data would violate the principles of meta-analysis, increase the risk of bias, and compromise reliability. Therefore, low-threshold analysis was not performed.

This meta-analysis demonstrated a pooled adjusted specificity of 0.875 for the FAST score, which was slightly lower than the previously reported value of 0.90, potentially due to differences in methodology and study populations (21). Bivariate model analysis yielded an adjusted specificity of 0.881 for the MAST score, showing a small drop relative to the MRI-based literature value (0.90) but still indicating strong diagnostic performance (19). Notably, continuity correction (+0.5) was used to reduce potential bias from extreme values (such as very high true negatives or very low false positives), which could be a reason for the slightly lower specificity compared with the original studies. Furthermore, the fact that most patients in our group were from Asia (66.7%) suggests that liver disease characteristics specific to that region might affect the generalizability of the thresholds. While the MAST score showed minor theoretical advantages, its use in clinical settings has two major limitations. First, it requires MRI technology, which is not widely available in many small clinics, necessitating careful evaluation of cost-effectiveness. Second, the positive predictive value (65% at the ≥ 0.67 cutoff) implies that 35% of positive results would still require biopsy confirmation. The FAST score was advantageous because FibroScan machines are more accessible; however, their performance depends on the operator’s skill. The MAST score may be most useful in hospitals with substantial resources, and implementation plans should consider the available equipment in each setting. We proposed a sequential diagnostic approach: tertiary care hospitals may use MAST first, while small clinics can use FAST for early checks and refer patients to larger hospitals if indicated. Because of its high specificity (87.5%), the FAST score is more suitable for “confirming” high-risk populations-that is, FAST-positive patients have a higher likelihood of the disease. However, due to its low sensitivity (44.7%), a negative FAST result cannot rule out disease. The MAST score was slightly more sensitive numerically (55.9%) and has better stability. It can be used for secondary evaluation of high-risk populations, but a 44% false-negative rate implies that negative results still cannot definitively exclude the disease; liver biopsy is still the gold standard. Neither score is suitable as an independent screening tool, and both are more suitable for use in combination within risk stratification pathways. Continuity correction (+0.5) helped resolve model instability due to zero-cell events, and the bivariate modeling approach captures the natural link between sensitivity and specificity. Although the sample size was limited, bivariate modeling and continuity correction were employed to minimize small-sample bias, providing a robust foundation for model selection. By sequentially excluding studies, we confirmed that our results were consistent, with no single study having a significant impact on our findings (ΔAUC <2%). Quality bias also showed minimal change (< 5% difference in effect size) when we excluded high-quality studies. Significantly, this study represents the first systematic comparison of these scoring systems in an Asian population, and our results suggest that implementing BMI-stratified cutoff values could be beneficial. This hypothesis requires validation in future prospective studies with larger cohorts (34).

There are several considerations to be acknowledged when evaluating our results. First, the studies we analyzed primarily originated from large hospitals, and some did not clearly state whether they used blinding when assessing outcomes. This could result in the findings appearing more applicable to all clinical settings, such as a general practitioner’s office, than they actually are, and possibly introduce some bias in how outcomes were determined. Second, it was not always clear how much time had passed between the liver biopsy and the other tests, and we lacked individual-level data. This constrained our ability to adjust more thoroughly for other variables that could have influenced the results. The cohort sizes in the studies were limited, so it was difficult to perform detailed analysis for specific subgroups, and we could not categorize the results by sex or underlying disease cause. From a methodological standpoint, although different methods of calculation (such as bivariate and random-effects models) yielded largely similar results, there were minor between-method variations, which frequently occur in these types of systematic reviews. We addressed this by using multiple methods to verify key results. Furthermore, while the different thresholds used for diagnosis demonstrate that one must consider the particular context, additional checks we performed indicated that the primary results are stable. Owing to the limited number of included studies (n = 4), we were unable to reliably assess publication bias, which is a limitation of this meta-analysis. Despite these issues, we believe that we followed a rigorous protocol from the outset, conducted numerous checks, and used optimal methods to analyze the data, which ensures that our findings are as valid as possible.

Future research should focus on standardizing cutoff optimization, establishing population-specific thresholds for Asian populations, developing BMI dynamic adjustment methodologies, incorporating new biomarkers, investigating the diagnostic utility of combining MAST scores with elastography, conducting full multi-parameter analysis with machine learning models, correlating score variations with hepatic cirrhosis and hepatocellular carcinoma incidence, and carrying out quality-adjusted life-year economic evaluations (35). Furthermore, prospective validation of both scores in primary care settings and assessment of their integration with novel biomarkers (such as Type III Collagen Propeptide) are warranted (24).

## Conclusion

In this systematic review and meta-analysis, we directly compared the diagnostic efficacy of FAST and MAST scores on active fibrotic MASH for the first time. The results showed that the MAST score was slightly better than the FAST score in sensitivity and overall diagnostic accuracy. Specificity was similar between the two scores, but the difference was not statistically significant. A significant threshold effect was observed for the FAST score, and cutoff values should be carefully selected for clinical use. The MAST score showed better between-study stability. The positive likelihood ratio and DOR were higher for the MAST score; however, it is limited by the availability and cost of MRI equipment. The FAST score is easy to use and has a high equipment penetration rate, which is more suitable for primary screening and preliminary risk stratification. Both scores identify high-risk MASH patients with moderate-to-high accuracy. Equipment conditions, patient characteristics, and the accessibility of medical resources should be comprehensively evaluated during clinical selection. Future large-scale, multicenter prospective studies are needed to further optimize diagnostic thresholds across diverse populations and verify their cost-effectiveness.

## Data Availability

The original contributions presented in the study are included in the article/Supplementary material, further inquiries can be directed to the corresponding author/s.

## Footnotes

1
www.prisma-statement.org
