# Supplementary material for: MAST versus FAST for active fibrotic MASH: a meta-analysis supporting risk-stratified diagnostic pathways
Source: Front Med (Lausanne). 2026 Apr 21;13:1779292. doi: 10.3389/fmed.2026.1779292 (PMC13138932; doi:10.3389/fmed.2026.1779292)
Supplement: Supplementary file 1 [file Table_1.DOCX]

Table S1 Histological Definitions of Active Fibrotic NASH in Included Studies

| Study ID | Cohort / Country | Definition of Target Condition | NAS Cutoff | Fibrosis Cutoff | Consistent with "NAS ≥ 4 and F ≥ 2" |
| --- | --- | --- | --- | --- | --- |
| ShiQi 2024 | China | "At-risk" MASH = NAS ≥ 4 and fibrosis stage ≥ F2 | ≥ 4 | ≥ F2 | Yes |
| Seung 2024 | Korea | High-risk NASH = NASH with NAS ≥ 4 and clinically significant fibrosis (F ≥ 2) | ≥ 4 | ≥ F2 | Yes |
| Mazen 2022 | USA | Fibro-NASH = NASH with NAS ≥ 4 and fibrosis stage ≥ F2 | ≥ 4 | ≥ F2 | Yes |
| Kento 2023a | Japan | Active fibrotic NASH = NASH with NAS ≥ 4 and fibrosis stage ≥ F2 | ≥ 4 | ≥ F2 | Yes |
| Kento 2023b | Japan | Active fibrotic NASH = NASH with NAS ≥ 4 and fibrosis stage ≥ F2 | ≥ 4 | ≥ F2 | Yes |
| Kento 2023c | USA | Active fibrotic NASH = NASH with NAS ≥ 4 and fibrosis stage ≥ F2 | ≥ 4 | ≥ F2 | Yes |
| footnotes: NAS, NAFLD Activity Score; F, fibrosis stage; MASH, metabolic dysfunction-associated steatohepatitis; NASH, nonalcoholic steatohepatitis. All studies used the histological scoring system proposed by the NASH Clinical Research Network (Kleiner et al., 2005). The terms "at-risk MASH," "high-risk NASH," "Fibro-NASH," and "active fibrotic NASH" are used interchangeably across studies to refer to the same target condition: NAS ≥ 4 and fibrosis stage ≥ F2. | | | | | |
